# Strategies for fine-mapping complex traits

**DOI:** 10.1093/hmg/ddv260

**Published:** 2015-07-08

**Authors:** Sarah L. Spain, Jeffrey C. Barrett

**Affiliations:** Wellcome Trust Sanger Institute, Wellcome Genome Campus, Hinxton, Cambridge CB10 1HH, UK

## Abstract

Genome-wide association studies (GWAS) have identified thousands of robust and replicable genetic associations for complex disease. However, the identification of the causal variants that underlie these associations has been more difficult. This problem of fine-mapping association signals predates GWAS, but the last few years have seen a surge of studies aimed at pinpointing causal variants using both statistical evidence from large association data sets and functional annotations of genetic variants. Combining these two approaches can often determine not only the causal variant but also the target gene. Recent contributions include analyses of custom genotyping arrays, such as the Immunochip, statistical methods to identify credible sets of causal variants and the addition of functional genomic annotations for coding and non-coding variation to help prioritize variants and discern functional consequence and hence the biological basis of disease risk.

## Introduction

Genome-wide association studies (GWAS) have identified thousands of robust and replicable genetic associations for complex diseases. This success was made possible by harnessing linkage disequilibrium (LD), or pairwise correlation, between nearby genetic variants. A few hundred thousand ‘tagging’ single nucleotide polymorphisms (SNPs) efficiently capture a sufficient proportion of the common variation in the genome to identify loci associated with disease. The cost effectiveness of GWAS genotyping arrays using these tagging SNPs allowed sample sizes larger than ever before and facilitated the detection of loci with an unbiased, hypothesis-free study design. The drawback of this design is that most strongly associated variants are likely to be in LD with the causal variant, rather than have a biological function themselves. This has led to criticism that GWAS neither identify causal variants nor explain most of the genetic variation in the population (1).

Prioritization of variants within GWAS-associated regions is an important focus of current research to enable the conversion of statistical associations into target genes, which provide insight into disease biology. This process can be broadly broken into two steps. The first is to assign well-calibrated probabilities of causality to candidate variants, known as fine-mapping. The second step is to try to connect these variants to likely genes whose perturbation leads to altered disease risk by functional annotation. As the majority of associated variants do not change the protein coding sequence of genes, there is a temptation to label the gene nearest to the variant with the smallest *P*-value (the ‘lead SNP’) as most likely to be causal. However, the physical distance of a variant to a gene is not substantive evidence of causality. Several research efforts are focussed on improving the functional annotation of regulatory SNPs, including ENCODE, NIH Roadmap Epigenomics and Fantom5. These resources are complemented by studies (such as GTeX) to identify SNPs known to affect the expression level of a particular gene in a particular tissue, known as expression quantitative trait loci (eQTL).

**Figure 1.**
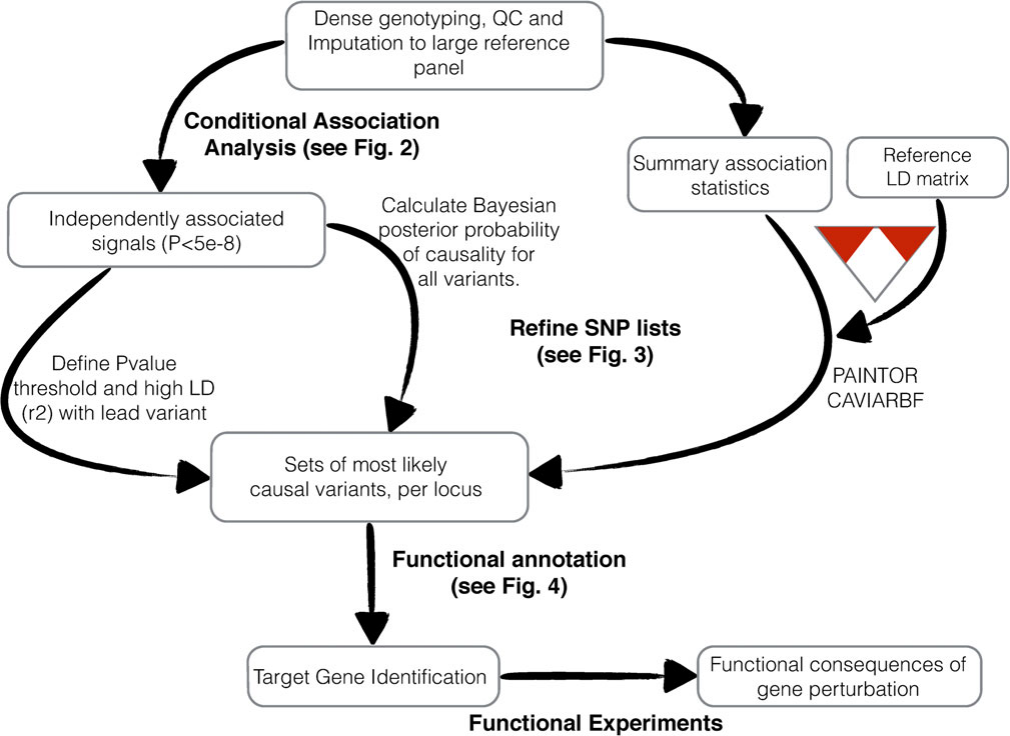
An overview of procedures for fine-mapping of GWAS loci.

In this review, we will discuss both statistical and functional fine-mapping efforts in the post-GWAS era, especially those using dense genotyping arrays, such as Immunochip and iCOGS. We will illustrate statistical methods that can be applied in a variety of circumstances and show how they can be connected to the functional annotation data sets described above. A generalized fine-mapping pipeline is depicted in Figure 1, showing the common steps to progress from associated variant to the identification of the potential causal gene. Box 1 summarizes the tools and databases mentioned in this review.

Box 1. **A tool box with URL for methods and annotation databases and tools.**

**Table:** 

Function	Tool	URL
Functional annotation of genetic variation	VEP	www.ensembl.org/info/docs/tools/vep/index.html
	ANNOVAR	http://annovar.openbioinformatics.org/en/latest/
Reference panels for imputation and LD estimation	1000 Genomes Project	http://www.1000genomes.org
Bayesian method to identify credible sets using genotype level data	BIMBAM	http://stephenslab.uchicago.edu/software.html#bimbam
Bayesian method optimized for trans-ethnic meta-analysis	MANTRA	Available by request from author (2)
Bayesian methods using summary statistics	CAVIARBF	https://bitbucket.org/Wenan/caviarbf
	PAINTOR	https://github.com/gkichaev/PAINTOR_FineMapping/
Bayesian methods including functional annotation	fGWAS	https://github.com/joepickrell/fgwas
	PICS	www.broadinstitute.org/pubs/finemapping/?q=home
Non-coding genome annotation projects	ENCODE	www.encodeproject.org
	Roadmap	www.roadmapepigenomics.org
	Fantom5	http://fantom.gsc.riken.jp
Databases using regulatory information to infer function	RegulomeDB	http://regulomedb.org
	HaploREG	www.broadinstitute.org/mammals/haploreg/haploreg.php
eQTLs	GTeX	www.gtexportal.org/home/
Enrichment analysis	Goshifter	www.broadinstitute.org/mpg/goshifter/
Drug target databases	ChEMBL	www.ebi.ac.uk/chembl/
	Drugbank	www.drugbank.ca
	Therapeutic target database	http://bidd.nus.edu.sg/group/cjttd/
Pubmed text mining of literature	GRAIL	https://www.broadinstitute.org/mpg/grail/
Protein–protein interactions	DAPPLE	http://www.broadinstitute.org/mpg/dapple/dapple.php
Pathway prioritization protocol	MEAGA	http://genome.sph.umich.edu/wiki/MEAGA

## Principles of Fine-mapping

Fine-mapping requires three essential components: (1) all the common SNPs in the region need to be genotyped or imputed with high confidence, (2) very stringent quality control and (3) large sample sizes to provide enough power to differentiate between SNPs in high LD.

To detect a GWAS signal, just one variant in LD is sufficient, but to accurately fine-map it requires information on all possible causal variants. Imputation methods, such as IMPUTE2 (3), MACH (4) and Beagle (5), together with the 1000 Genomes Project reference panels (6), fill in the gaps for variants that were not included on genotyping arrays. This allows the crucial assumption when evaluating the relative evidence of each associated SNP being causal that the true causal SNP is being considered (see below). Strict quality control procedures are paramount to the accuracy of imputation, to ensure that genotyping errors are excluded prior to imputation, usually by manually checking the intensity cluster plots for all associated variants. This is especially important in large meta-analyses where cases and controls may be genotyped in different centres and often must be performed more than once (7,8).

To increase power for fine-mapping, large international consortia were formed that combined their data sets and collaboratively designed custom genotyping arrays. These arrays, containing ∼200 000 variants, provide dense genotyping of previously discovered GWAS regions for fine-mapping. For instance, the Metabochip was designed by the Cardio-Metabochip Consortium (9) and focussed on associated regions for phenotypes including Type 2 diabetes (T2D), coronary artery disease and quantitative traits such as body mass index. Similarly, the Immunochip consisted of variants selected primarily from the GWAS-associated regions of 12 immune-mediated phenotypes (10). Recently, the COGS project brought together four consortia to design the iCOGS array to investigate the genetics of breast, prostate and ovarian cancers. These collaborations enabled large meta-analyses where all samples had been genotyped on the same chip, ideally suited for statistically fine-mapping association signals.

**Figure 2.**
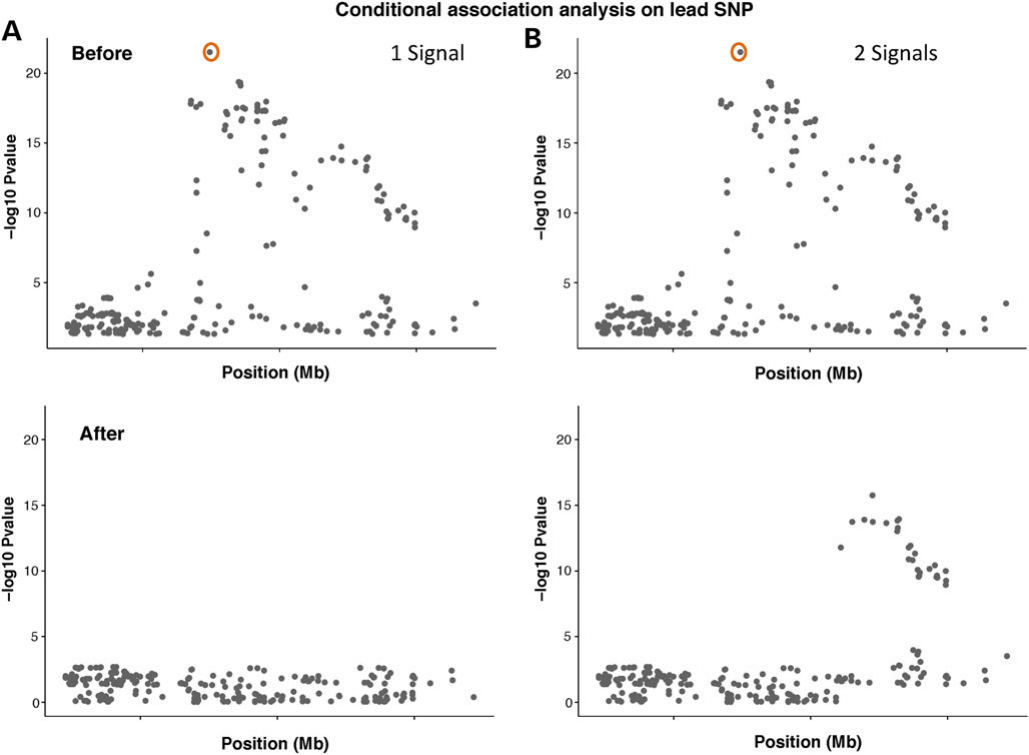
Illustration of conditional association analysis conditioning on the lead SNP, indicated by the orange circles (the SNP with the lowest *P*-value in the GWAS) using genotype level data for (**A**) one independent signal and (**B**) two independent signals. The top plots show the results of the association analysis and the bottom plots the result after conditioning on the lead SNP.

Fine-mapping studies typically impute from these dense chips to a suitably dense reference panel such as the 1000 Genomes Project (6), then perform association analysis and stepwise conditional analysis to identify independent signals within regions (Fig. 2). This process is crucial for downstream fine-mapping, as regions with multiple independent signals can interfere with each other in the statistical analyses described below.

## Statistical Methods for Fine-mapping

A number of different methods have been developed for the prioritization of causal variants to explain association signals. They can broadly be classified into two groups: triaging variants based on *P*-values or LD to the lead SNP and Bayesian methods that assign posterior probabilities of causality to each SNP.

A simple approach is to consider all SNPs with a *P*-value less than a certain threshold (e.g. 5 × 10^−8^, the standard for genome-wide significance), as candidates for causality. This is rarely sensible, as *P*-values are influenced by study-specific factors such as power (determined by sample size) and locus-specific factors such as minor allele frequency and effect size. Therefore, *P*-values that have been calculated in different studies with different power have different implications for the plausibility of a true association and are not necessarily comparable (11). A slightly more sophisticated approach involves considering all SNPs above a certain LD threshold with the lead SNP as potentially causal. Although this is less arbitrary than *P*-value thresholds, it still ignores the properties of the study or locus, as higher power can differentiate SNPs in higher LD.

In a Bayesian framework, the evidence for association at each variant is measured using a Bayes Factor, which, with certain assumptions, can be used to calculate the posterior probability for each variant of being causal for the association in that region (11,12). These posterior probabilities are the ratio of evidence for each variant versus all others, which makes it a useful comparator for fine-mapping purposes (12). Assuming there is exactly one causal variant in a region, and all variants are included in the analysis, then for any ‘credible set’ of variants, we can state that the causal variant will be included in the set with confidence equal to the sum of the posteriors of the SNPs in the set. Many different programmes for fine-mapping have been developed to produce credible sets of causal variants (13–17) and are summarized in Box 1.

**Figure 3.**
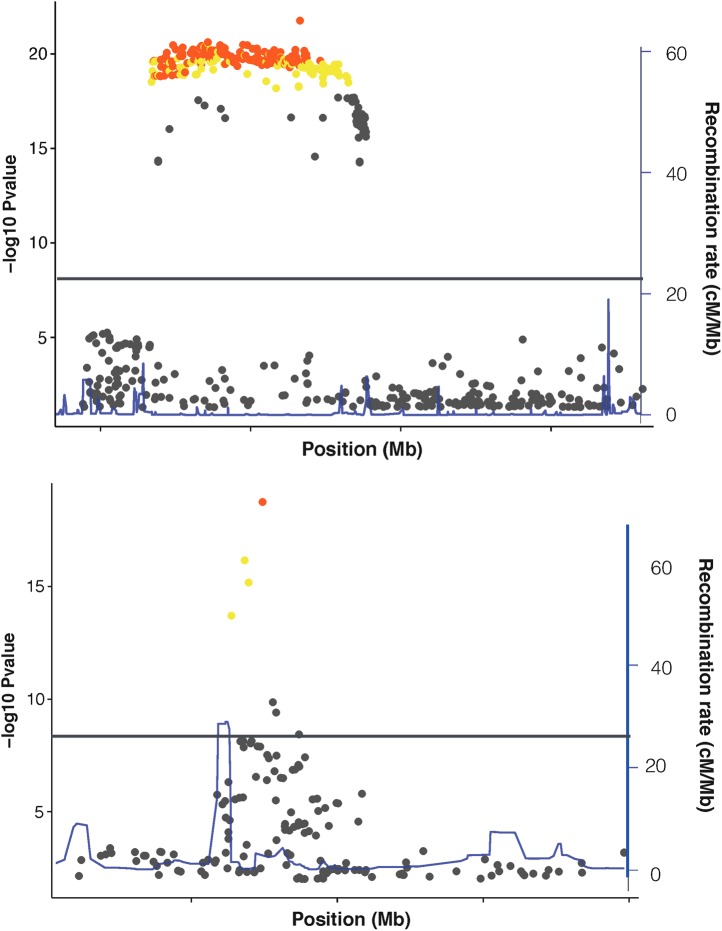
Fine-mapping from many variants in an associated region to a credible set of most likely causal variants. The plots illustrate the associated variants under two potential GWAS association peaks, with −log10 *P*-value plotted against the chromosome position. The grey lines indicate the position of genome-wide significance at 5 × 10^−8^, showing the number of variants that would be prioritized by *P*-value alone. The points plotted in yellow are the variants in high LD (*r*^2^) with the lead variant. The points coloured in orange are the variants included in the 95% credible set for the most likely causal variants.

Figure 3 shows a comparison of these three approaches. Some regions can be refined to a handful of variants (or indeed just one), whereas other credible sets will contain hundreds of variants. In the latter case, although the method has not improved the prioritization of causal variants much, there is still useful information to be gained from the proportion of posterior probability assigned to each variant, especially if a few variants account for a large fraction. Bayesian posteriors can be directly compared between variants, either within the same study or across different studies, which can be key in the context of necessarily large international collaborations in complex disease genetics. Additionally, compared with approaches based on *P*-values, in a Bayesian analysis, it is straightforward to weight evidence for a given variant by incorporating prior knowledge of functional annotation or consequence (for example fGWAS and PAINTOR, discussed below).

The methods described above rely on raw genotype data, which are not always available (Fig. 1). Therefore, several approaches have been developed to attempt to identify independent associations in the same region, and construct credible sets for these associations, from summary statistics alone. This is particularly useful for meta-analyses of separate data sets, where genotype level data may not be available. A recent analysis (18) compared BIMBAM (which can incorporate multiple causal variants but requires genotype level data) with two methods, CAVIARBF [a modified implementation of CAVIAR (19)] and PAINTOR (14), which require only the summary test statistics and a matrix of the pairwise correlation coefficients (*r*^2^) of the variants in each associated region, which could be from a population matched reference panel. Performance was measured by the proportion of causal SNPs identified from the results of an analysis of 100 simulated data sets from a continuous trait. Where the number of causal variants is 1, all three methods perform similarly, suggesting that summary statistic methods are valuable for this scenario. However, when considering simulations with two or more causal variants, CAVIARBF outperformed the other methods. These simulations may be more favourable to summary statistic approaches than real applications where the reference LD matrix does not exactly match the populations studied in the GWAS data, and further direct comparisons will be necessary to explore how well they can fine-map regions with multiple independent signals. Furthermore, without full conditional analyses from raw genotypes, a parameter must be specified for the number of causal variants, and it has been demonstrated that setting this value to 1 can impair the performance for cases where there are multiple causal variants (14).

## The Prioritization of Variants Using Functional Annotations

**Figure 4.**
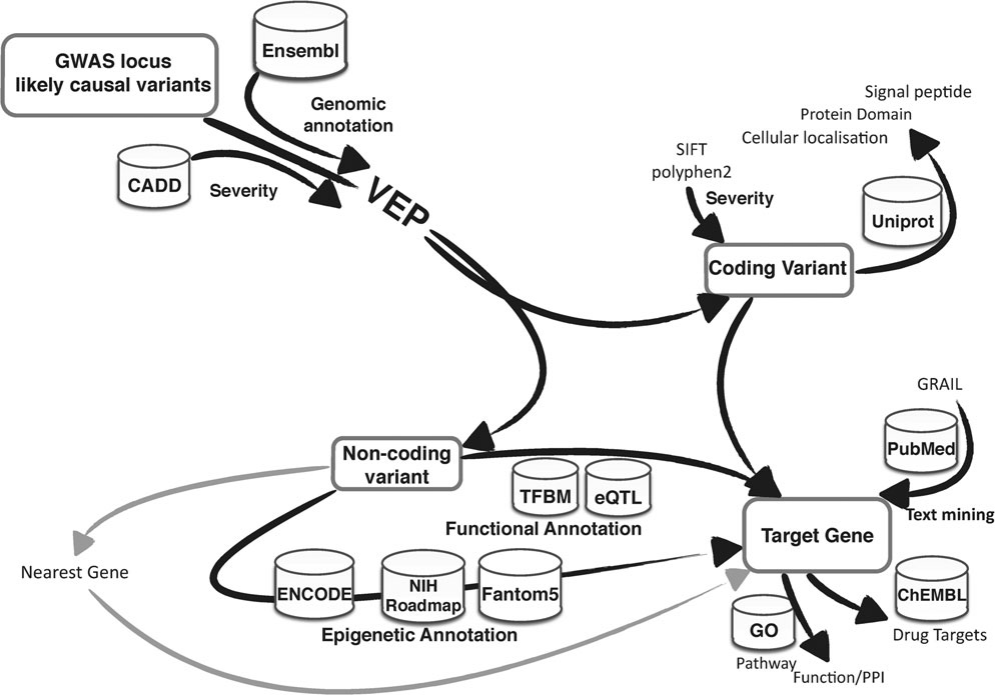
Functional annotation schematic illustrating the annotation possibilities in the process of associated variant to target gene mapping. VEP, variant effect predictor; CADD, Combined Annotation-Dependent Depletion; TFBM, transcription factor binding motif.

The availability of tools to easily annotate genetic variants by genomic location and potential functional consequence has greatly aided causal inference for variants identified in GWAS and fine-mapping studies. These approaches complement the statistical techniques described above, as they bring independent sources of information about what each variant is likely to do biologically, rather than how strongly the genetic evidence supports it. The Ensembl variant effect predictor (VEP) (20), which has a web interface and stand-alone functionality, and ANNOVAR (21) can both be easily incorporated into analysis pipelines. These tools have the ability to incorporate variant annotations from various databases and resources, including coding and regulatory regions. Scores to predict the deleteriousness of the variants can also be included such as polyphen2, SIFT (for coding variation) and CADD (which includes all variants) (see Fig. 4).

Although protein coding variants are easiest to build a case for prioritization and to design experiments to assay functional consequence, they account for a small fraction of GWAS hits (22). Projects focussed on understanding the function of the non-coding genome started primarily with the Encyclopedia of DNA Elements (ENCODE) project, which sought to describe the functional elements of the human genome (23). ENCODE contains information about methylation and chemical modifications to histones and the binding activity of transcription factors (TFs) and the DNA elements that regulate genes. Two subsequent consortia, functional annotation of the mammalian genome (FANTOM) and the NIH Roadmap Epigenomics Consortium (24), have sought to extend this work and assign further functional annotations to the genome. FANTOM5 expands the original mouse FANTOM project (25) to build models of the transcriptional regulation across all cell types in the human body using Cap Analysis of Gene Expression, which assays RNA expression. This work provided maps of transcripts, TFs, promoters and enhancers that were active across the different cell types (26,27). The NIH Roadmap Epigenomics Consortium (24) focussed on the mapping of DNA methylation, histone modifications and chromatin accessibility using cell lines and primary human tissues. The data provide information on the regulatory elements controlling gene expression in 127 tissues and cell types, including healthy and disease affected samples. Modified histone residues are markers of chromatin structure and function, which are associated with regulation of transcription (24). The locations of these modifications can be used to indicate whether a genetic variant is affecting this process in some way and is thus useful in determining the functional importance of associated variants. Changes in the higher level structure of chromatin can also affect gene expression. Experimental methods, such as chromosome conformation capture (3C) (28), and various protocols of HiC [which offers higher resolution and increased scale of interaction testing (29)] can infer this 3D structure of chromatin. These methods can be used to determine the presence of physical interactions between promoters and possible enhancer sequences (30). Some fine-mapping studies have used 3C-based methods to confirm the evidence from enhancer mapping to identify likely target genes (31,32).

The information provided by these projects link associated variants to a phenotype via an understanding of the biological basis of these associations in terms of disease pathology and ultimately identify causal genes. These data can be combined with statistical fine-mapping in multiple ways. Several approaches, such as fGWAS (16), use functional annotations as priors for potential causal variants. The PAINTOR method (14) described above can perform similar re-weighting in analysis of summary statistics. This approach can also determine which annotations are most informative and identify associations that do not meet stringent significance levels based on genetic data alone. Enrichment analyses are used to determine whether the identified variants, or subsets of variants, are significantly enriched for overlaps with regulatory regions of the genome. Programmes, such as Goshifter (33), have been developed to test the significance of these enrichments while controlling for confounding effects of correlated annotations and LD.

## Bringing Together Statistical and Functional Fine-mapping

Many studies are now successfully applying the entire workflow shown in Figure 1 to go from associated locus to implicated gene (Table 1). For instance, Farh *et al.* (15) applied a method called probabilistic identification of causal SNPs (PICS) to disease-associated loci from 21 autoimmune diseases. They combined these data with an improved epigenetic map of cis-regulatory elements for immune cell (34). The variants from the credible sets were mapped to this epigenetic map and revealed that 60% of the most likely causal variants mapped to enhancer elements, especially those activated in stimulated CD4+ T cells. There was also enrichment for coding variation (14% of predicted causal variants), DNase hypersensitivity sites and TF-binding sites from ENCODE (23), including NF-Kβ and IRF4. Specific examples of fine-mapped signals include a Crohn's disease risk variant located in the intron of *SMAD3* (rs17293632, C>T) that prevents the robust binding of AP-1, which in turn disrupts AP-1 regulation of TGF-β–SMAD3 pathway and highlights a potential mode of action to increase disease risk. An eQTL analysis refined variants within the *IKZF3* locus in multiple sclerosis to two variants with independent effects on the expression of *IKZF3*. One decreased expression and increased risk of MS, rs12946510, and the other, rs907091, increased expression had no effect on risk of MS. The mechanism by which rs12946510 decreased expression of *IKZF3* was unclear from the sequence alone and this example serves to illustrate that the effect of expression changes on disease risk is context dependent and not always straightforward to interpret.

**Table 1.** Selection of fine-mapped GWAS loci from recent studies, concentrating on large studies using the custom genotyping chips

Phenotype	References	Target gene	Array	Method (further details)
T1D	Onengut-Gumuscu *et al.* (31)	AFF3	Immunochip	Bayesian analysis and 3C
		BCAR2		
		PTPN22		
		IFIH1		(three independent signals)
		CTSH		
		TYK2		(two independent signals)
		FUT2		
T2D	DIAGRAM Consortium *et al.* (35)	JAZF1	Metabochip	TE meta, MANTRA (overlaps enhancer region)
		SLC3OA8		(coding variant, functional experiment)
LDL cholesterol	Musunuru *et al.* (36)	SORT1	–	siRNA knockdown (functional experiment)
Crohn’s disease	Farh *et al.* (15)	SMAD3	Immunochip	PICS, TF binding
Multiple sclerosis		IKZF3	Immunochip	PICS, eQTL analysis
		IL2RA		PICS, enhancer map
Breast cancer	Ghoussaini *et al.* (37)	IGFBP5	iCOGS	Enhancer map (variant flanks transcriptional enhancer and interacts with promoter)
	Dryden *et al.* (32)	IGFBP5	–	CHi-C
Breast cancer	French *et al.* (38)	CCND1	iCOGS	3C, allele-specific CHiP
Breast cancer	Orr *et al.* (39)	KLF4	Additional genotyping	*P*-value, LD and functional annotation (enhancer element, two independent signals)
Breast cancer	Meyer *et al.* (40)	FGFR2	iCOGS	DHS (variants alter TF binding)
Prostate cancer	Kote-Jarai *et al.* (41)	TERT	iCOGS	Chip-seq and expression analysis
Breast cancer Ovarian cancer	Bojesen *et al.* (42)	TERT	iCOGS	mQTL TCGA (decreased methylation levels increase cancer risk)
RA	Okada *et al.* (43)	CDK6 /CDK4	Immunochip	TE meta, *P*-value, LD and functional annotation (known drug targets for cancer)
Psoriasis	Tsoi *et al.* (44), Das *et al.* (45)	IL13	Immunochip	Functional SNP in high LD with lead SNP
Psoriasis	Tsoi *et al.* (44)	TRAF3IP2	Immunochip	Functional lead SNP
		STAT2		Functional SNP in high LD with lead SNP
		PRSS53		Functional SNP in high LD with lead SNP
		CARD14		Functional lead SNP
		TYK2		Functional lead SNP
		YDJC		Functional SNP in high LD with lead SNP
		ERAP2		Functional SNP in high LD with lead SNP
Psoriasis	Tsoi *et al.* (44)	NFKBIZ	Immunochip	eQTL
		FUT11		eQTL
		MYOZ1		Expression analysis (psoriatic versus normal skin)
		NFKBIZ		

The target gene is the gene implicated by the functional annotation or fine-mapping analysis. TE = trans-ethnic meta-analysis; TF = transcription factor; 3C = chromosome conformation capture; CHi-C = capture Hi-C, similar to 3C; DHS = DNAse hypersensitivity sites; RA = rheumatoid arthritis.

A recent Type 1 diabetes (T1D) study (31) refined 50 susceptibility regions using a combination of Bayesian methods (12) to identify 99% credible sets and functional enrichment analysis using data from the ENCODE (23) and NIH Roadmap Epigenomics projects (24). The results showed significant enrichment of credible set SNPs in enhancer chromatin states in the thymus, CD4+ and CD8+ T cells, B cells and CD34+ cells. The results suggest that variation in enhancer sequences is relevant to T1D risk. Focussing on the credible set SNPs that were annotated as functional (missense, from VEP, or that were located within enhancer regions), the authors highlighted 29 SNPs in 12 loci with small credible sets (<5 variants) that improved evidence for several candidate genes (*PTPN22*, *IFIH1*, *CTSH*, *TYK2* and *FUT2*). The combination of small credible sets with improved annotation of non-coding consequences substantially reduces the size of the associated region and number of plausible target genes.

In breast, ovarian and prostate cancers, multiple independently associated variants in the *TERT* locus were identified, using the iCOGS array (41,42), including evidence of an association between rs10069690 and two tumour methylation probes that are less methylated with the cancer risk allele. Additionally, Kote-Jarai *et al*. (41) found, in a functional follow-up study, that rs2242652 (correlated with rs10069690) increased expression of *TERT*. Chromatin conformation assays were also used to refine the 11q13 locus associated with breast cancer to *CCND1* (38) and the 2q35 locus to *IGFBP5* (32).

In the rheumatoid arthritis (RA) fine-mapping analysis (43), the authors performed a three-stage meta-analysis and developed an annotation pipeline (see Fig. 4) to identify target genes after prioritizing likely causal variants, which identified 98 candidate genes from within 101 risk loci. The prioritization pipeline scored variants based on annotations including missense variant, cis-eQTL in peripheral blood mononuclear cells, T cell/monocytes and potential target genes using pubmed text mining (Grail), protein–protein interactions (Dapple), primary immunodeficiency, somatic mutation, mouse knockout phenotypes and molecular pathway analysis. The final step was to compare candidate genes to the known drug target genes where the authors found a significant enrichment for approved RA drugs (*P* = 0.0035), including an anti-IL6R (tocilizumab) and a JAK3 inhibitor (tofacitinib). They also showed that drugs approved for other phenotypes, such as those that target CDK6 and CDK4 for cancer treatment, may also be applicable to RA. This analysis demonstrates the utility of this process to aid the potential repurposing of drugs for the treatment of different phenotypes (43).

## Trans-ancestry Meta-analysis for Fine-mapping

Most of the approaches discussed so far assume association analyses in relatively homogenous populations, with consistent patterns of LD, as this scenario is most straightforward for discovery association analysis. Meta-analysis of samples from many different ancestries can be challenging when attempting to discover associations, but ancestral differences in LD can be advantageous when attempting to fine-map. In 2011, a Bayesian method, called MANTRA, was developed to enable trans-ancestry meta-analysis to overcome this problem of LD heterogeneity between different ancestries (2). If an associated allele is shared between groups with different ancestry, the differences in LD between populations, such as European and African, can be useful to help fine-map the locus by restricting the credible set of variants to those that are in LD with the causal variant in all populations (2). Although multiple independent signals in disease-associated loci have been demonstrated, conditional analysis in this context is not straightforward due to the differences in LD between populations in the study (35).

A trans-ethnic meta-analysis, utilizing MANTRA, was used to refine the credible sets of causal variants for 10 loci associated with T2D (35). The authors performed a meta-analysis of GWAS from Asia, Mexico and Europe incorporating 26 488 cases and 83 964 controls using Metabochip genotypes imputed to 1000 Genomes Project haplotypes. Two of the loci with functional consequences highlighted in the article were *JAZF1* and *SLC3OA8*. The credible set for *JAZF1* locus was reduced to four SNPs (16 kb), through the trans-ancestry method. One of these SNPs, rs1635852, was shown (using ENCODE data) to reside in a region of open chromatin with enhancer activity. Multiple TFs bind to the region and the SNP shows allelic differences in enhancer activity in pancreatic islet cells (46), highlighting its potential importance in T2D pathogenesis. The *SLC3OA8* (Zn28) locus was refined to a credible set of two SNPs in the analysis, one of these, rs13266634 (Trp325Arg), which is the lead SNP in the trans-ethnic meta-analysis, is non-synonymous and has an established mode of action. *SLC3OA8* is a Zn2^+^ transporter and in 2009, Nicolson *et al*. (47) demonstrated that the Arg325 variant allele exhibits reduced transporter activity than the wild-type allele, suggesting that Zn2^+^ transport is important in T2D risk and thus highlighting a potential therapeutic intervention for the condition.

## Conclusion

After the success of the GWAS approach to identify regions of the genome significantly associated with hundreds of different diseases, a major current challenge is to translate those findings into causal variants and target genes. As the majority of associated variants are in non-coding regions of the genome, improved functional annotation for these variants is essential. Projects like ENCODE, NIH Roadmap Epigenomics and FANTOM5 have begun to make advances in characterizing regulatory regions. Advances in the confident identification of causal variants from GWAS identified regions have also been made, with multiple different Bayesian methods to calculate posterior probabilities of causality for each variant in a locus allowing between- and within-study comparisons to be made. The ability to incorporate functional or other annotations to weight causal probabilities and programmes that only require summary statistics offer additional flexibility of analytical approach. The ultimate value of GWAS will come from informed biological inferences on causal mechanisms, aided by pinpointing causal variants and target genes. This will enable more accurate pathway and functional analysis and facilitate the understanding of disease biology and identification of drug targets to help ameliorate the symptoms of complex diseases.

*Conflict of Interest statement*. None declared.

## Funding

J.C.B. is funded by the Wellcome Trust grant WT098051. S.L.S. is funded by the Centre for Therapeutic Target Validation. Funding to pay the Open Access publication charges for this article was provided by Wellcome Trust Grant WT098051.
